# A bibliometric analysis of fungal volatile organic compounds

**DOI:** 10.1186/s40694-025-00203-x

**Published:** 2025-07-02

**Authors:** Kustrim Cerimi, Dierk-Christoph Pöther, Stefanie Klar

**Affiliations:** Federal Institute for Occupational Safety and Health, Unit 4.II.2 Bioaerosols, Nöldnerstraße 40-42, Berlin, 10317 Germany

**Keywords:** Fungal volatile organic compounds, fVOCs, Bibliometric analysis, Inter-organismic communication, Biotechnology, Biofumigation

## Abstract

**Background:**

Fungal volatile organic compounds (fVOCs) serve as crucial mediators in ecological interactions and hold significant potential for applications in agriculture and biotechnology. Fungi establish inter-organism communication through volatile molecules, enabling them to regulate plant growth and interact with diverse soil-dwelling organisms. This study integrates a comprehensive literature survey and bibliometric analysis to capture the complexity and interdisciplinary nature of fVOC research, drawing on PubMed, Google Scholar, and Scopus databases spanning 2000 to 2023.

**Results:**

The findings highlight the role of fVOCs as essential chemical messengers in inter-organismic communication, their contribution to sustainable agricultural practices as plant growth promoters, and their significance in human sensory perception, particularly in culinary contexts. Our bibliometric analysis of 3,738 publications maps fVOC research trends worldwide using co-occurrence and -citation analyses. The latter uncovered an early research focus on yeast fermentation and antimicrobial activity, which has since expanded to sustainable agriculture, biofumigation, endophytic fungi, and the development of advanced analytical techniques. Emerging research clusters focus on plant–fungus communication, the biotechnological production of aroma compounds, and the influence of fVOCs on human sensory experiences.

**Conclusions:**

The fVOC research field has matured during the last two decades. Promising avenues for future exploration include the improvement of crop resilience, the advancements of eco-friendly technologies, such as biological pest management or VOC-driven fertilisation, and a better understanding of the intricate volatile communication that drives fungal interactions with other kingdoms of life.

**Supplementary Information:**

The online version contains supplementary material available at 10.1186/s40694-025-00203-x.

## Introduction

Fungal organisms produce a diverse range of distinctive odours detectable by humans and many other organisms, including plants and animals. These odours consist of organic compounds that can easily evaporate due to their chemical and physical properties at 25 °C, with relatively high vapour pressures and low boiling points. Volatile organic compounds can be classified into three main categories based on their physical properties: very volatile organic compounds (VVOCs), volatile organic compounds (VOCs) and semi-volatile organic compounds (SVOCs) which have higher molecular weight and boiling points than VOCs, making them less likely to become vapors at room temperature [1]. Growing fungi are shown to produce VOCs as a mixture of compounds of many molecular sizes in which the types, the numbers, and the amounts of individual VOCs are variable and the complex cocktail varies temporarily and changes with temperature, substrate, and other environmental variables for each species [2]. Most volatile molecules have a small molecular mass of less than 300 kDa and are mainly lipophilic [3]. These compounds are commonly referred to as volatile organic compounds (VOCs), microbial VOCs (mVOCs), or, more specifically, fungal VOCs (fVOCs). The dictionary definition for describing something volatile as a state of matter is “characterised by a natural tendency to disperse into smoke or vapour” and the term “volatilome”, which encompasses the entire spectrum of volatile metabolites within the metabolome, has recently gained attraction in many studies [4–6]. Due to their chemical properties, fVOCs can disperse from their point of origin through the atmosphere, soil, and liquids [7], where they serve as semiochemicals for intra- and inter-organismic communication between various organisms, such as bacteria, fungi, and plants [8]. The kingdom of *Fungi* is genetically and morphologically very diverse and includes millions of species, ranging from uni- to multicellular organisms, colloquially known as yeasts, moulds, mushrooms, or lichens. They play critical roles in ecosystems as decomposers, mutualists, and pathogens [9]. The fungal kingdom is the second largest group of species after insects, with an estimated 2–3 million species and currently 160,000 accepted species [10]. There are more than 200 orders of fungi which have been classified into 12 phyla and 6 different subkingdoms [11]. However, newer works even suggest 19 different fungal phyla [12]. Fungi are ubiquitous and typically found in terrestrial and marine environments. Due to their versatile metabolic capabilities, many fungi synthesise a variety of fVOCs, allowing them to communicate with all domains of life and form intricate interactions across kingdoms and species. As such, they drive key ecological processes and are vital for all ecosystems [13]. The research community studying fVOCs is rapidly growing and highly interdisciplinary, spanning several disciplines from genomics, microbiology, ecology, biotechnology, up to medicine. Fungal VOCs and other fungal metabolic products are of huge interest to different industries and harnessed as cell factories of food, feed, pharma, chemical, biofuel, textile, and building applications [14, 15].

We aimed in this study to fully explore the environmental and industrial impact of fungal VOCs. We harnessed bibliometric data available in several public databases and established a bibliometric network. Our aim was to identify major research trends and findings within the growing fVOC community and identify potential connections between different disciplines and research fields by mapping and clustering bibliometric data. Such bibliometric network approaches have recently been harnessed for several publications, ranging from business-to-business marketing [16], software marketing [17, 18], and software development [19], as well as publications in the life sciences, for example, for identifying trends in circular RNA [20], COVID-19 [21, 22], and fungal pathogenicity [20, 23, 24]. However, they have not yet been applied for searching the field of mVOCs and fVOCs. Given the growing body of studies and the significance of mVOCs and fVOCs for our understanding of ecological processes and the evolution of ecosystems, a thorough assessment of the evolution of both scientific fields is needed. We, thus, provide a general overview of the mVOC research field in this study, but with a particular focus on fVOCs. This allowed us to identify past, current and future research trends and key research labs that are currently involved in fVOC science.

## Methods

### Literature review and screening

A comprehensive literature review (Table 1) was conducted for the introduction and subsequent sections, utilising databases such as PubMed, Google Scholar, and Scopus. Various keyword combinations were used to capture a broad spectrum of publications across disciplines relevant to fVOC research. Selected references were incorporated into the results sections to support key findings on the ecological roles of fVOCs in inter-organismic communication, their economic and biotechnological applications, and their distinct significance in human perception and culinary contexts.

**Table 1:** Keywords used for the bibliometric survey

Category	Search strings
Volatile organic compounds (VOCs)	mVOC*, fVOC*, volatile organic compound*, fungal volatile organic compound*
Fungi and related	Fungi*, mushroom*, indoor fungi*, yeast*

The literature search was performed on 14 Arpil 2024. A wildcard (*) was used to include different spellings of the keywords in the search

### Bibliometric analysis

The bibliometric analysis (Table 2) was conducted using VOSviewer (version 1.6.20), a freely available science mapping tool [25, 26]. Scopus, a comprehensive scientific database (https://www.scopus.com/), served as the primary source for this analysis. Four distinct search queries were used to capture a broad spectrum of publications related to fVOC research.

**Table 2:** Search queries for the bibliometric analysis with vosviewer. Data was retrieved on 14 April 2024

Number of query	Search query
1	(TITLE-ABS-KEY(fung* W/10 (“volatile organic compound*” OR VOC* OR volatile* OR mVOC* OR fVOC* OR “microb* volatile compound*”))) AND PUBYEAR > 1999 AND PUBYEAR < 2024
2	(TITLE-ABS-KEY(mold* W/10 (“volatile organic compound*” OR VOC* OR volatile* OR mVOC* OR fVOC* OR “microb* volatile compound*”))) AND PUBYEAR > 1999 AND PUBYEAR < 2024
3	(TITLE-ABS-KEY(yeast* W/10 (“volatile organic compound*” OR VOC* OR volatile* OR mVOC* OR fVOC* OR “microb* volatile compound*”))) AND PUBYEAR > 1999 AND PUBYEAR < 2024
4	(TITLE-ABS-KEY(mushroom* W/10 (“volatile organic compound*” OR VOC* OR volatile* OR mVOC* OR fVOC* OR “microb* volatile compound*”))) AND PUBYEAR > 1999 AND PUBYEAR < 2024

The Scopus database operators were used to obtain a comprehensive range of publications. The latter were screened for keywords within the title, abstract, and author keywords. Four primary terms: “fungus”, “mold”, “yeast”, and “mushroom”, were used in combination with keywords related to VOCs, such as “fVOC” or “volatile organic compounds” (Table 2, Numbers 1–4). The near operator (W/10) was applied, allowing for a maximum of ten words between any two terms, to capture closely related content. Publications from the period between 2000 and 2023 were examined.

Publications were initially exported directly from Scopus along with complete metadata (including references) into plain text files, which were subsequently processed using Excel. The publications were manually sorted by date to count annual publication frequencies, providing insights into changes in the total number of publications over the period chosen (see supplementary files).

Regarding the bibliometric mapping, nodes represent entities such as publications, journals, or researchers, while edges indicate relationships between them. The strength of these relationships was quantified by the “total link strength” metric, calculated within VOSviewer. Node relationships were also visualised based on relative distances, with closely positioned nodes reflecting stronger connections. The specific algorithms used to determine node locations and link strengths are not discussed in detail here, as the co-occurrence and co-citation analysis methods followed Van Eck and Waltman [27].

A co-occurrence analysis of author keywords was conducted on pooled search queries for current research trends up to the end of 2023. Historical trends were assessed through co-occurrence analysis in five-year intervals, except for the last three years. Additionally, a co-citation analysis was performed on data from the entire search period to identify key contributors and influential works. This analysis examined the interconnections among research articles by assessing how often they were cited together, focusing on the 20 most influential works.

## Results

The results of the literature search covering publications from 2000 to 2023 were manually screened for research articles relevant to the ecological roles of fVOCs in inter-organismic communication, their economic and biotechnological applications, and their significance in human perception and culinary products. The pooled search queries for the bibliometric analysis led to a total of 3,738 publications (Table 3), which were analysed using VOSviewer.

**Table 3:** Number of fVOC publications published in the period 2000–2023

Query search term	No. of publications
*Fung*	2,065
*Mold*	203
*Yeast*	1,212
*Mushroom*	258

### Fungal VOCs as chemical messengers in inter-organismic communication

Fungal VOCs are produced during the primary and/or secondary metabolism of uni- and multicellular fungi. They exhibit a wide range of structural variations and functional groups, including aliphatic alcohols, cycloalkanes, aldehydes, esters, ketones, benzenoids, terpenes, and naphthalene derivatives, to name but a few [28]. They play essential roles in inter-organism communications and influence plant behaviour through the inhibition of pathogen growth, the activation of plant immunity, or by improving plant tolerance to abiotic stress [29]. It is often root structures, either primary or lateral roots, which are affected by the presence of soil volatile compounds below ground. It has been shown that these compounds may restructure the proteome mechanism in plant roots [30] and that fVOCs, such as dimethyl disulfide, can decrease primary root synthesis and increase lateral root formation in plants, as a recent study has shown [31]. The VOC emissions from fungi such as *Trichoderma viridae* or *Fusarium oxysporium* have been shown to boost root development in general [32]. Apart from the influence on the root system, fVOCs from root endophytic fungi can also have other plant-promoting effects. Some VOCs, such as 3-octanol or 1-octen-3-ol produced by *Trichoderma* species, can effectively inhibit seed germination [33], and fungi of the genus *Serendipita* spp. can enhance photosynthetic efficiency, increasing Calvin cycle intermediates and, subsequently, raising sugar concentrations [34]. By contrast, fVOCs emitted by fungal pathogens can also suppress plant growth and actively weaken host plants. *Fusarium culmorum* VOCs, for instance, can alter the nutritional status of plants from a distance, influencing the micronutrient content, including Fe, Zn, Cu, and Mo [35]. Whether an fVOC has a plant-promoting or -inhibiting effect is not predetermined and it may be difficult to classify an fVOC as phytotoxic, because one and the same compound can have contrasting results on plant growth behaviour [36]. Not only are plants affected by the variety of fungal volatiles, but also other organisms below ground, such as nematodes. The basidiomycete *Pleurotus ostreatus* has been proved in a study to effectively kill a nematode such as *Caenorhabditis elegans* through toxocyst structures containing the fVOC 3-octanone, which paralysed the nematode [37]. Other fungal VOCs affecting nematodes, such as cyclohexanamine, cyclohexanone, and cyclohexanol from the fungus *Duddingtonia flagrans*, also exist [38]. Fungi and bacteria which inhabit soil can also interact through VOC emissions, influencing microbial behaviour. A study demonstrated that fVOCs can induce specific phenotypic responses in bacteria, affecting growth, enhancing antimicrobial activity, promoting biofilm formation, and altering motility [39]. Endophytic fungi produce a plethora of different fVOCs [40, 41], such as one major group of volatiles, sesquiterpenes, which have gained a lot of attention in the last few years as contributors in plant-microbe interaction [42]. A study has shown that these volatiles affect the motility of *Serratia marcescens* and induced a growth reduction in *Pseudomonas fluorescens* [43].

### Fungal VOCs as compounds of economic interest

Fungal VOCs have important applications in biotechnology and agriculture, since the dependence on chemicals in agriculture in the growing demand for food may create an ecological imbalance or result in ecotoxicity [44]. Different fungal species can be used as biocontrol agents, including the *Trichoderma* species, arbuscular mycorrhizas, yeasts and endophytes, and their biocontrol mechanisms include not only antibiosis but also competition to other pathogens or even mycoparasitism [45]. Among the variety of fungal organisms, *Trichoderma* species are mainly studied for their ability to either enhance plant growth or protect plants against potential pathogens by fVOCs. A comprehensive review has summarised many examples of how various *Trichoderma* species, such as *T. harzianum*, *T. atroviridae*, *T. koningi* or *T. gamsii*, are influencing plants by growth simulation, biomass accumulation, delaying the negative effects of droughts, or improving root branching [46]. On the other hand, the volatilome of *Trichoderma* species is known to be useful in the protection against plant-pathogens. Various candidates, such as *T. asperellum*, *T. harzianum*, *T. atroviride*, or *T. virens*, show the growth inhibition of potential phytopathogenic organisms [47]. One of the most harmful fungi in crops and agriculture is *Fusarium oxysporum*, which causes vascular wilt diseases in economically important crops throughout the world [48]. It has even been listed among the one the of ten most important fungal plant pathogens [49]. *Trichoderma* spp. has been shown to be able to suppress *Fusarium*, which, for instance, causes wilt disease in tomato plants [50]. The concept that both *Trichoderma* and *Fusarium* species may interact and produce several VOCs in response to each other has also been discussed [51]. Additionally, fVOCs can function as biofungicides, as they often inhibit the growth of both plant-pathogenic fungi and bacteria [52]. Fragrances and aromas have traditionally been obtained through the extraction of plant- or animal-derived materials for centuries. Chemical-based methods replaced these traditional techniques during industrialisation in order to meet the growing demand for fragrances and odour-based products. Additionally, microbial and fungal may play a direct role in production processes, with biotechnology offering an attractive means of generating fragrant and flavoursome molecules on a large scale [53]. Many fungi, such as *Ceratocystis* spp. and *T. viridae*, can produce fruity or floral odours, including coconut-flavoured lactone, 6-pentyl-alpha-pyrone, and floral- flavoured terpenes, suh as citronellol and linalool. Vanillin, an important food additive with a distinctive aroma, has been produced through a two-step fermentation process involving the ascomycete *A. niger* and the basidiomycete *Pycnoporus cinnabarinus* [54]. Biotechnological approaches are becoming increasingly important in the production of bio-based aromas and scents, agriculture, and crop science. Fungal VOCs also hold great potential for enhancing industrial processes and driving innovative biotechnological solutions.

### Fungal VOCs as compounds for human diet

Humans have a strong connection to odours of microbial or fungal origin. This form of interspecies communication follows fundamental principles, such as avoiding danger, for example, detecting rotten food, while intentionally using and promoting specific odours beyond just food. According to a recent experimental setup, up to 110 different VOCs could be detected in the initial stage of food decay depending on temperature [55]. In addition, the natural decay of food caused by fungi or other microbes was recently used to investigate neural processes involved in distinguishing between edible and inedible food items [56].

The characteristic odour of rotten fruit arises from a mixture of VOCs emitted either by the metabolic machinery of the fungi itself and/or as a direct result of the food degradation process. The VOCs such as 1-Octen-3-ol, 3-octanone, benzenes, benzaldehyde, 1-methoxy-3-methylbenzene, and 1-methyl-4-propan-2-ylbenzene – released by fungi including *F. oxysporum*, *Cladosporium* sp., *A. niger*, *Mucor plumbeus*, *Trichoderma* spp., and *Penicillium expansum* – accumulate as food decay processes, particularly in the later stages of infection [57]. Additionally, fungi can emit VOCs, such as 3-chloroindole or indole, which are often perceived as highly unpleasant or even faecal-like [58].

Fungal odours play a crucial role in human nutrition, particularly in culinary contexts. Some of the most intensely aromatic foods and mushrooms belong to the genus *Tuber* spp., commonly known as truffles. The latter have been used since ancient times by civilisations such as the Babylonians, Etruscans, Egyptians, Greeks, and Romans. Their distinctive and highly prized flavour makes them one of the most expensive culinary ingredients, often referred to as “underground gold” or the “diamond of the kitchen” [59]. According to a recent study, up to 60 different compounds were identified by headspace solid-phase microextraction from different known tuber species, such as *Tuber aestivum*, *Tuber borchii*, or *Tuber nitidum* [60].

Among the best-known truffle species, *Tuber melanosporum*, *Tuber aestivum*, and *Tuber borchii* can produce up to 75 different VOCs [61]. Certain volatile compounds, such as dimethyl sulphide, dimethyl disulphide, 3-(methylthio) propanal, 2-methylbutanal, 3-methylbutanal, 2-methylbutan-1-ol, 3-methylbutanol, and oct-1-en-3-ol, contribute to the characteristic fungal aroma of truffles [59, 62].

Notably, dimethyl sulfide has been identified as the primary attractant for dogs and pigs, which are traditionally used to locate truffles in the wild [63, 64]. This attraction is believed to be an evolutionary strategy of truffle fungi, as the VOCs diffuse through the soil, drawing animals that dig up the fungi and aid in spore dispersal. Additionally, the volatilome of truffles has been discussed in relation to its potential aphrodisiac properties for humans [65]. However, compared to fresh truffles, storage conditions and duration can significantly influence the overall volatilome, possibly due to interactions with the associated microbiome of the truffle [66].

### Trends in fungal VOC science

A bibliographic analysis was conducted to identify research trends and provide a historical overview to analyse and visualise the broad scope of fVOC research. As has been outlined in the methods section, various search queries were applied in the scientific database *Scopus* (Table 2, Pos. 1–4). Data were collected over a 23-year period (2000–2023).

**Fig. 1:**
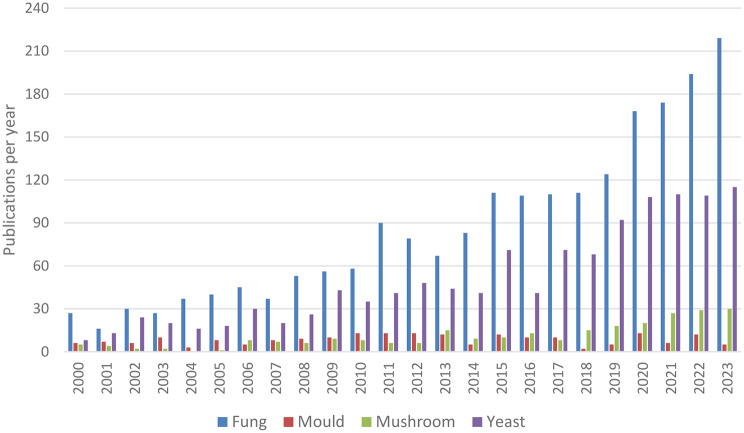
Annual publication counts in fVOC science among search terms and overall publication from 2000 to 2023

The search queries highlight specific aspects of fVOC research, each targeting particular terms. On the whole, interest in fVOC science has steadily increased over the past 23 years. However, growth has varied across subfields; publications related to keywords such as “fungi” and “yeast” have risen significantly, whereas topics such as “mushroom” and “mold” have shown only modest growth (Fig. 1).

#### Co-occurrence analysis and interdisciplinary clusters in fVOC research

**Fig. 2:**
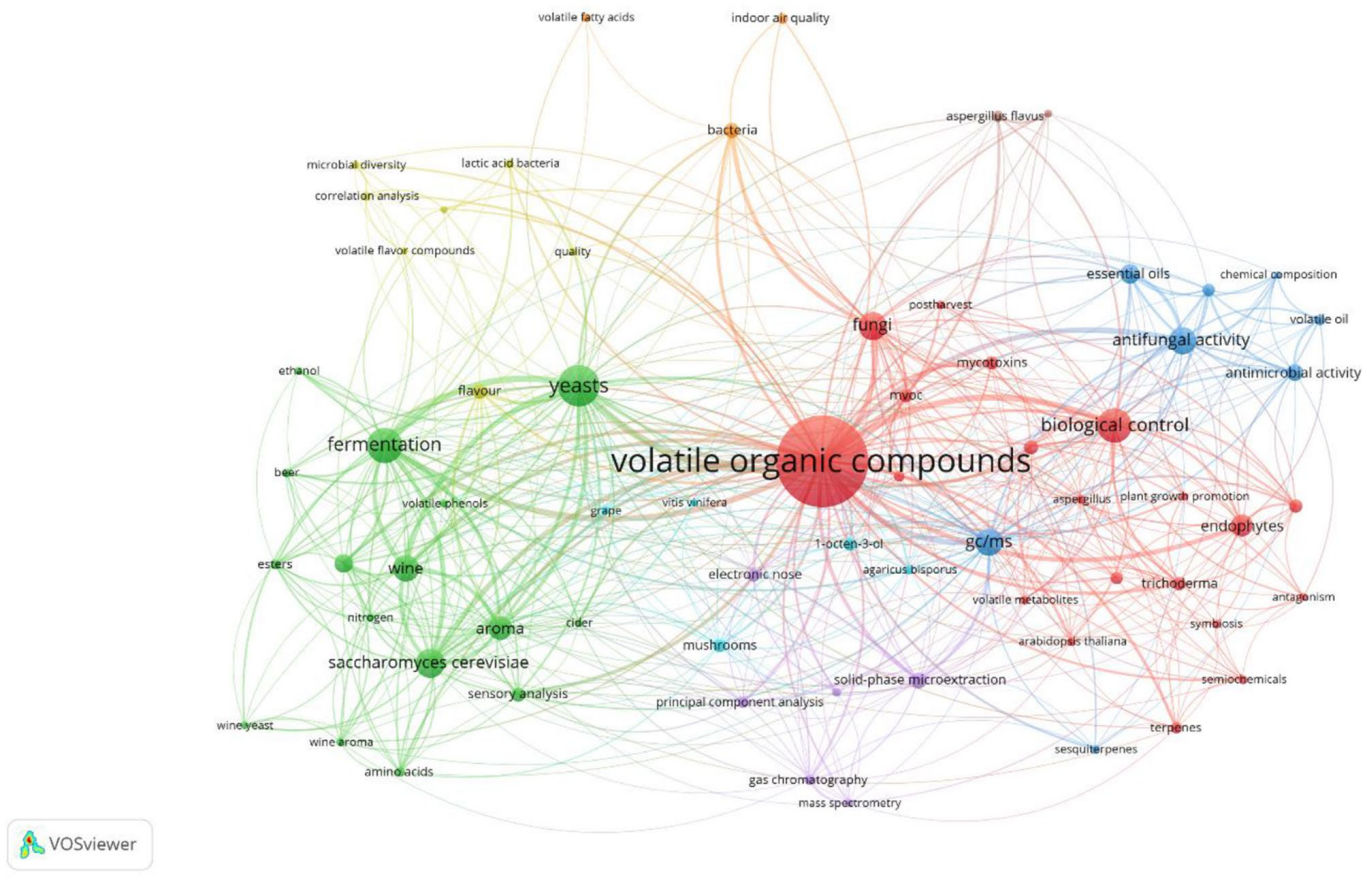
Network visualisation of the author keyword co-occurrence analysis of the pooled search queries. The VOSviewer determined several clusters from 67 author keywords separated by different colors

As has been described in the methods section, the VOSviewer co-occurrence analysis visualises keywords in fVOC research, with nodes representing terms and lines indicating their relationships. Thicker lines represent stronger connections, illustrating the interdisciplinary nature of fVOC science. Based on keywords provided by the authors, the network revealed seven distinct but interconnected clusters (Fig. 2). The green cluster focuses on fermentation, yeast, *Saccharomyces cerevisiae*, and the wine industry, featuring terms such as “esters”, “ethanol”, and “wine yeast”, highlighting a strong presence of food and beverage research. Terms such as “yeasts” and “*Saccharomyces cerevisiae*” were prominent, indicating a specific interest in yeast over broader fungal research.

Meanwhile, the purple cluster focuses on analytical techniques, highlighted by terms such as “solid-phase microextraction”, “electronic nose”, and “gas chromatography”, which are predominantly used in fVOC detection and analysis. The ecological roles and plant interactions of fVOCs are represented by the red cluster, indicated by keywords such as “endophytes”, “*Trichoderma*”, and “*Arabidopsis thaliana*”, underscoring the natural roles of fVOCs in ecosystems and plant health. The yellow cluster includes keywords such as “lactic acid bacteria” and “quality”, suggesting a focus on microbial contributions to flavour and quality in fermented products, linking microbial community dynamics to food science. The blue cluster emphasises antimicrobial properties and applications, including the terms “essential oils”, “antifungal activity”, and “*Aspergillus flavus*”. This cluster reflects interest in using fVOCs for biocontrol and antimicrobial purposes. Lastly, the orange cluster connects to health and indoor air quality concerns, shown by terms including “indoor air quality” and “bacteria”.

This co-occurrence analysis generally demonstrated the broad and interconnected nature of fVOC research. The variety of clusters revealed the interdisciplinary scope within this field, with a significant overlap between ecological, industrial, analytical, and health-related topics. This complex structure highlights the integrative nature of fVOC science, emphasising collaboration across multiple domains. Having outlined the overall structure of fVOC research over the past 23 years, the following analysis examines how these topics have evolved over specific time periods. We can gain insight into the emergence and development of key research areas, as well as identifying how certain topics have grown or shifted focus within the field, by exploring time trends. This temporal analysis provided a clearer picture of the dynamic nature of fVOC research and its progression over time.

#### Development of trends in the spectrum of fVOC research

**Fig. 3:**
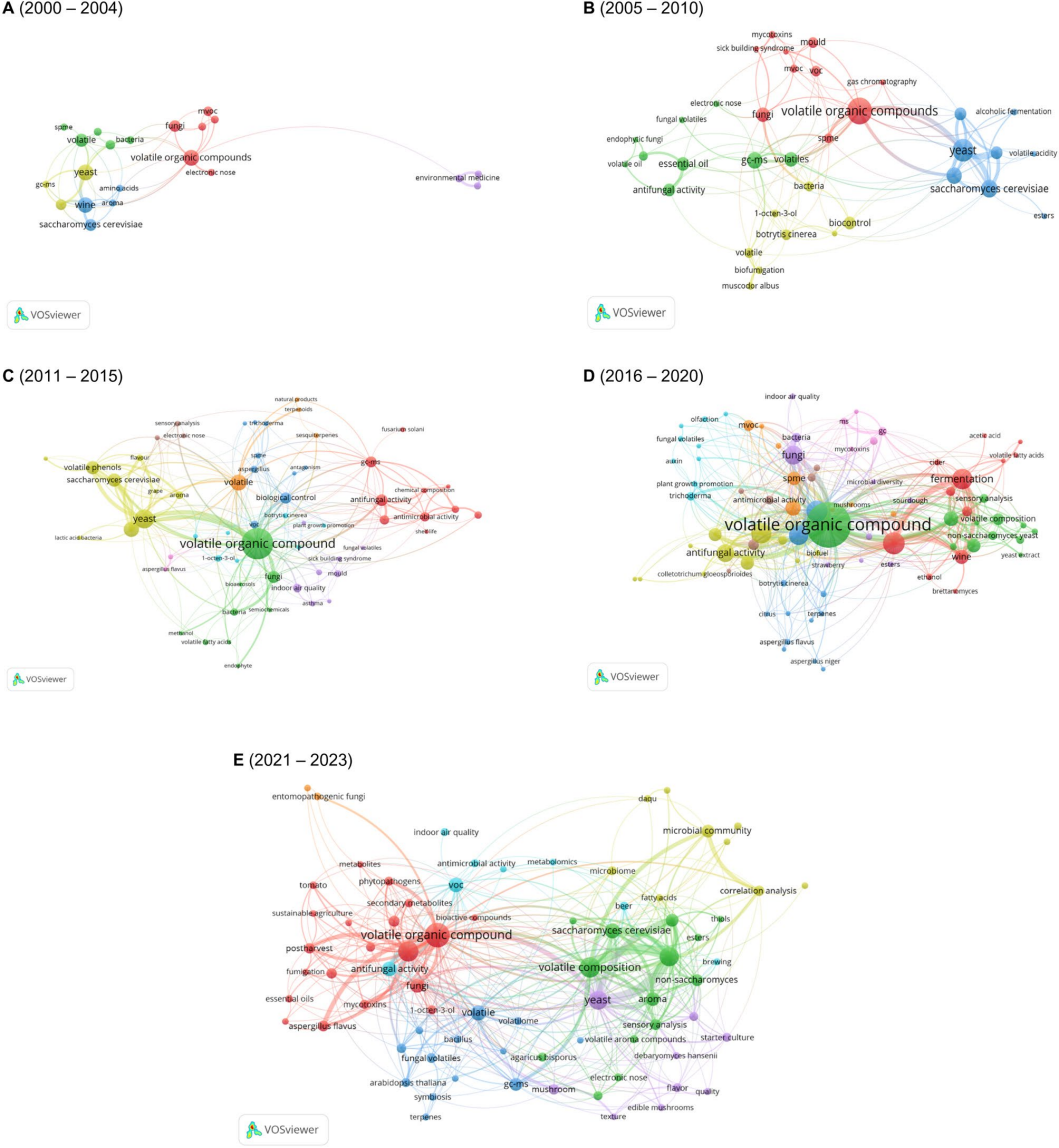
Network visualisations of the co-occurrence analysis using VOSviewer based on author keywords within specific time ranges. Co-occurrence analysis was performed for the periods 2000–2004 (**A**), 2005–2010 (**B**), 2011–2015 (**C**), 2016–2020 (**D**), and 2021–2023 (**E**)

The thematic landscape of fVOC research has evolved over the past two decades from a small, focused domain into a rich, interconnected field spanning multiple disciplines. At the beginning of the period from 2000 to 2004 (Fig. 3, A), the co-occurrence network centred around key terms including “volatile organic compounds” and “yeast”, with connections extending to related terms such as “fungi”, “bacteria”, and “wine”. Early research interest in VOC detection technology was also shown by terms such as “fungi” and “electronic nose”. The co-occurrence network for 2005–2010 (Fig. 3, B) showed increased complexity, with more prominent terms and connections compared to the previous timeframe. “Volatile organic compounds” were closely linked with “fungi” and “yeast”, later including associations with “sick building syndrome”, “mycotoxins”, and “mold”, reflecting a growing focus on health-related impacts. Compared to the previous period, where only some keywords were associated with microbial interactions, later periods saw related terms such as “antifungal activity”, alongside “fungal volatiles” and “endophytic fungi”, becoming more prominent, indicating an interest in the ecological and biocontrol roles of fVOCs. Moreover, terms such as “biofumigation” and “*Botrytis cinerea*”, represented by the yellow cluster, suggested an agricultural application focus not present previously. Fermentation processes remained a key research focus until 2010, as indicated by the prominence of terms such as “yeast” and “*Saccharomyces cerevisiae*”, alongside wine-related terms including “alcoholic fermentation” and “volatile acidity”.

An increased diversity in terms connecting “volatile organic compound” to a broader range of topics was evident in the period 2011–2015 (Fig. 3, C). Moreover, a new field became prominent, with terms such as “indoor air quality”, “sick building syndrome”, “mold”, and “asthma” within the purple cluster, indicating an early interest in health-related topics and the possible association of fVOCs with health problems. The network also revealed more specialised yet persistent clusters, such as the prominent yellow cluster focusing on “yeast” and “*Saccharomyces cerevisiae*”. The bioactive properties of fVOCs and their environmental impact, indicated by terms including “antifungal activity” and “antimicrobial activity”, were more widely interconnected with other fields compared to the previous period. Analytical methods, such as “gas chromatography-mass spectrometry ”, were positioned as bridging technologies with broad interconnections to other clusters. This period generally reflected the growing complexity and specialisation in fVOC research, with distinct areas of interest in biocontrol, health impacts, and industrial applications.

In addition to clusters already established before 2015, several interconnected topics emerged between 2016 and 2020 (Fig. 3, D), resulting in a network with a main field and well-defined sub-areas in biotechnological applications, food science, and ecological research, highlighting a growing interest in their applications, particularly in agriculture and crop science. Alongside the broader research topic of fermentation processes, another sub-area was the antifungal activity of fVOCs, indicating interest in their use, for instance, in agricultural or crop science.

The co-occurrence network for the final years 2021–2023 (Fig. 3, E) revealed an increasingly dense web of connections around the term “volatile organic compounds”, reflecting a highly diverse and interconnected research landscape. However, compared to 2016–2020 (Fig. 3, D), the sub-areas appeared to be slightly more distinct, forming clearer clusters. One cluster centred around “volatile composition”, with links to “*Saccharomyces cerevisiae*”, “volatile composition”, and “non-saccharomyces yeasts”, indicating ongoing research in fermentation, brewing, and aroma, emphasising food science and flavour chemistry. Conversely, ecological and agricultural applications of fVOCs – particularly in postharvest treatments and pathogen management – were clustered around “volatile organic compound”. While fermentation research focused on the composition of various fVOCs, crop science emphasises their plant-promoting or -inhibiting roles, highlighted by terms such as “antifungal activity”, “fungi”, “fumigation”, and “sustainable agriculture”. Lastly, the purple cluster related to sensory applications, featuring terms including “electronic nose” and “quality”, reflected advances in analytical methods for evaluating flavour and aroma in food products. This network highlighted the maturity of fVOC research, with strong interdisciplinary connections across agriculture, food science, and environmental studies.

Fungal VOC research has transformed from a niche focus on microbial interactions and fermentation into a complex, interdisciplinary field over the last 23 years. Early studies centred on yeast, antimicrobial activity, and fundamental health effects. The scope expanded by the mid-2000s to include agricultural applications, such as biocontrol and the development of advanced analytical methods for detecting and characterising fVOCs.

By the 2010s, fVOC research had expanded to include plant health, indoor air quality, and biofuels, leading to the emergence of distinct clusters on fermentation, antifungal properties, and ecological interactions. The field has become even more interconnected in recent years, incorporating topics such as sustainable agriculture, microbial communities, and food science. This evolution highlights the increasing versatility of fVOC research, with broad applications in agriculture, medicine, and environmental science.

#### Mapping knowledge patterns in fVOC science: insights from a Co-citation analysis

**Fig. 4:**
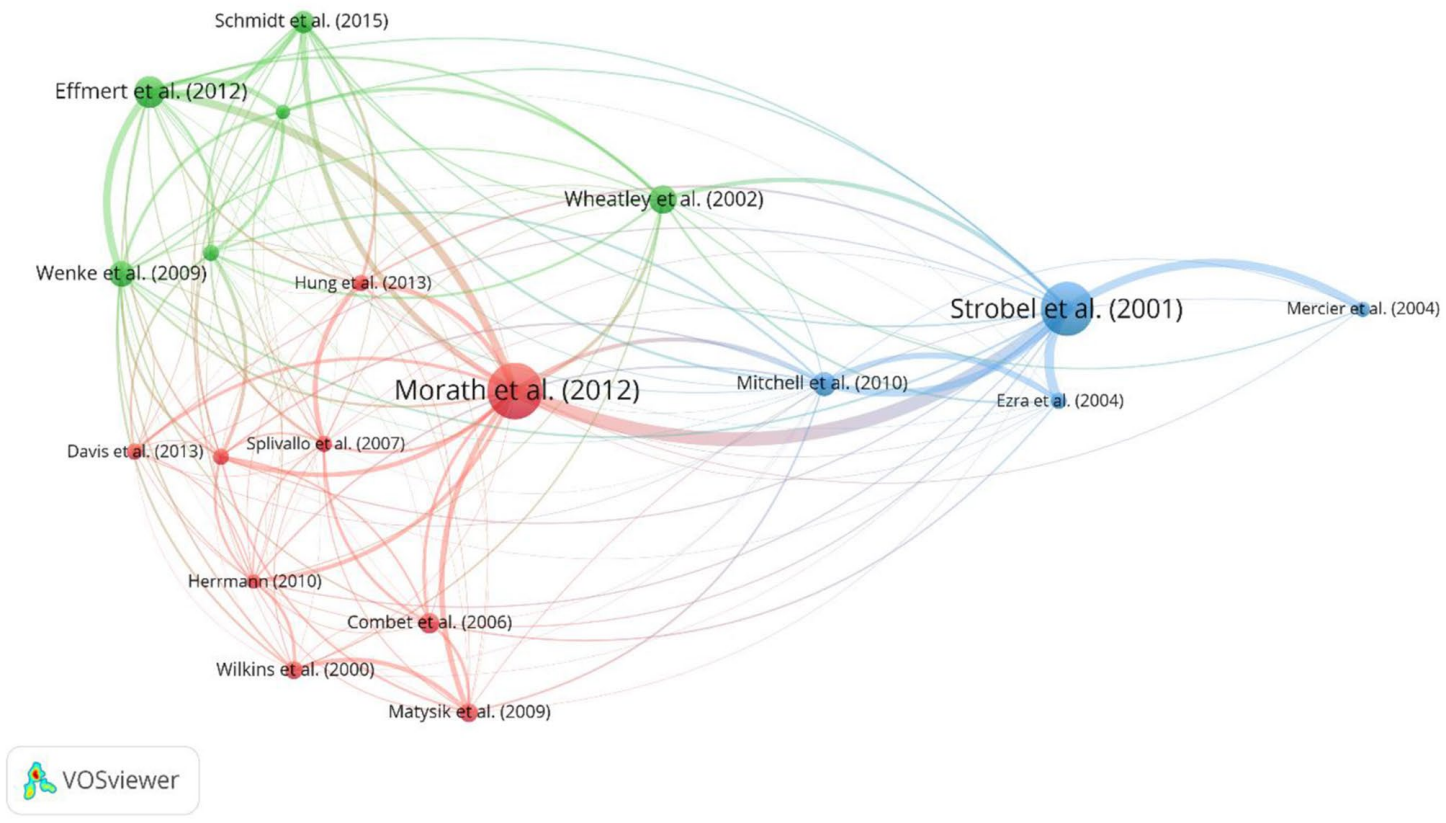
Network visualization of the co-citation analysis using VOSviewer, based on the pooled search terms, highlighting the network of the 20 most influential works identified in our literature review

A co-citation analysis was conducted using VOSviewer to explore the relationships between documents by assessing how often they are cited together. This approach helped to identify influential studies and provided insights into the knowledge structure and thematic connections within fVOC research (Fig. 4).

The co-citation network analysis highlighted the broad and interdisciplinary nature of fVOC science, with certain publications attracting significant attention. Larger nodes, such as those representing Morath et al. (2012) and Strobel et al. (2001), indicate highly influential works frequently cited in other studies. The connecting lines represent co-citation links, with thicker lines signifying a higher frequency of co-citations between specific publications, suggesting closer thematic relationships. While clusters represent broad thematic groups, strong co-citation connections – illustrated by thicker lines – can also exist within them, reflecting overlapping research interests.

The network is divided into distinct clusters, each summarising works that were frequently cited together. Each colour represents a thematic cluster, highlighting the diversity of research areas within fVOC science (Table 4).

**Table 4:** The most influential works based on a co-citation network analysis in vosviewer. The number of co-citations and total link strength resulted directly from the vosviewer.software. Results are sorted based on their total link strength

Position	Publications / Year	Co-citations	Co-citations per year (until 2023)	Citations [Total]	Total link strength	DOI (URL) / Reference
1	Morath et al. (2012)	84	7.63	378	56	10.1016/j.fbr.2012.07.001 [67]
2	Strobel et al. (2001)	80	3.63	435	50	10.1099/00221287-147-11-2943 [68]
3	Effmert et al. (2012)	45	4.09	383	35	10.1007/s10886-012-0135-5 [69]
4	Mitchell et al. (2010)	34	2.61	165	25	10.1099/mic.0.032540-0 [70]
5	Wenke et al. (2010)	36	2.76	213	23	10.1007/s00425-009-1076-2 [71]
6	Schmidt et al. (2015)	31	3.85	324	22	10.1038/ismej.2015.42 [72]
7	Korpi et al. (2009)	23	1.64	404	20	10.1080/10408440802291497 [73]
8	Wheatley (2002)	40	1.90	240	20	10.1023/A:1020592802234 [74]
9	Ezra et al. (2004)	23	1.21	204	18	10.1099/mic.0.27334-0 [75]
10	Kai et al. (2009)	20	1.42	383	18	10.1007/s00253-008-1760-3 [76]
11	Schulz et al. (2007)	23	1.43	/	18	10.1039/B507392H [77]
12	Hung et al. (2013)	23	2.30	138	17	10.1016/j.funeco.2012.09.005 [78]
13	Splivallo et al. (2007)	22	1.38	147	17	10.1111/j.1469-8137.2007.02141.x [79]
14	Herrmann (2010)	20	1.54	/	16	10.1002/9780470669532 [80]
15	Matysik et al. (2009)	26	1.86	77	16	10.1016/j.chemosphere.2009.02.010 [81]
16	Combet et al. (2006)	29	1.71	206	13	10.1007/S10267-006-0318-4 [82]
17	Wilkins et al. (2000)	25	1.09	119	12	10.1016/S0045-6535(99)00273-8 [83]
18	Mercier & Jiménez (2004)	21	1.11	190	10	10.1016/j.postharvbio.2003.08.004 [84]
19	Davis et al. (2013)	23	2.30	272	9	10.1007/s10886-013-0306-z [85]
20	Pretorius (2000)	30	1.30	782	0	https://doi.org/10.1002/1097-0061(20000615)16:8<675::AID-YEA585>3.0.CO;2-B [86]

The large blue cluster within the co-citation network highlights research on the antimicrobial properties of VOCS produced by various *Muscodor* species. Studies by Strobel et al. (Table 3, Pos. 2), Ezra et al. (Table 3, Pos. 9), and Mitchell et al. (Table 3, Pos. 4) focused on how VOCs emitted by these endophytic fungi can inhibit or eliminate a range of fungal and bacterial pathogens. Notably, *Muscodor albus* and *Muscodor crispans* produce a complex mixture of volatile compounds effective against diverse pathogens, underscoring their potential in controlling both plant and human diseases. These studies explored the ecological role of *Muscodor* spp., showing how these fungi colonise plant tissues and enhance host defence through VOC production. This naturally occurring antifungal and antibacterial mechanism suggests possible applications in mycofumigation, where fungal volatiles are harnessed to manage microbial infections in agriculture. One recent example is their use against the banana pathogen *Mycosphaerella fijiensis*, as described by Mitchell et al. (Table 3, Pos. 4).

Additionally, Strobel et al. (Table 3, Pos. 2) demonstrated that fVOCs of *M. albus* and the overall fungi, as a mycofumigant, can have other practical uses. When exploring different *Muscodor* isolates, especially in the study by Ezra et al. (Table 3, Pos. 9), there was also an indication of the adaptability of the fungus, as strains from various host plants exhibit distinct volatile profiles and antimicrobial spectra. The potential applications described in the works within the blue cluster generally indicated a strong interest in leveraging fVOCs as a natural antimicrobial agent, spanning agriculture, medicine, and industry.

The next large cluster (red) in the co-citation network included studies such as Morath et al. (Table 3, Pos. 1), Hung et al. (Table 3, Pos. 12), and Splivallo et al. (Table 3, Pos. 13). This group primarily investigated the interactions between fVOCs and plants, focusing on both the beneficial and inhibitory effects these compounds can have.

Morath et al. (Table 3, Pos. 1) provided a comprehensive overview of fVOCs, highlighting their diverse roles in ecological interactions, particularly in biocontrol and signal transduction. This work also explored the biotechnological potential of fVOCs in applications such as biofuel production and mycofumigation. The broad scope of this research may explain its central position and large size within the co-citation network, as it spans multiple research areas. The specific growth-promoting effects of VOCs from *Trichoderma viride* on *Arabidopsis thaliana* were the central focus in a paper by Hung et al. (Table 3, Pos. 12). This study demonstrated that these VOCs can significantly enhance plant biomass and chlorophyll concentration without direct contact, underscoring the influence of fVOCs on plant physiology and their potential for agricultural applications. Meanwhile, Splivallo et al. (Table 3, Pos. 13) investigated the inhibitory effects of VOCs produced by truffles (*Tuber* species) on *A. thaliana*, noting that these compounds can inhibit root growth and induce oxidative stress. This work added another dimension to the understanding of fVOCs by showing that, depending on the fungal species and context, these compounds can also affect plant health detrimentally. Ongoing research into the ecological significance of fVOCs and their potential for practical applications in agriculture and biotechnology focused largely on works within the red cluster.

Lastly, the green cluster in the co-citation network included works by Effmert et al. (Table 3, Pos. 3) and Wenke et al. (Table 3, Pos. 5), both exploring the role of mVOCs in ecosystems below ground. These studies emphasised the deep interactions and interspecies communication facilitated by volatiles between various soil organisms, including bacteria, fungi, plant roots, and other soil inhabitants. Effmert et al. (Table 3, Pos. 3) provided an extensive review of mVOCs, detailing how these compounds mediate a complex network of inter- and intraspecies communication within soil environments. This work catalogued over 800 known mVOCs and discussed how these emissions influence microbial behaviours and interactions. The study highlighted the ecological significance of mVOCs in soil, suggesting that they play a key role in maintaining soil microhabitat balance and facilitating communication below ground. Similarly, Wenke et al. (Table 3, Pos. 5) addressed the dynamics between volatiles below ground, focusing on their role as semiochemicals that mediate interactions between plant roots and soil organisms. The review described how these volatile compounds act as chemoattractants or -repellents and co-ordinate communication among plants, fungi, bacteria, and other organisms living in the soil. This study underscored the importance of soil volatiles in understanding the integrity of ecosystems by examining both eukaryotic and prokaryotic organisms in these interactions. Taken together, these works emphasised the fundamental function of mVOCs in soil ecology. They illustrated how these volatiles serve as communication signals that support interactions and contribute to ecosystem stability, particularly in the complex environment below ground.

The clusters of the co-citation network in fVOC research reveal three main topics thematically that have been established over the last two decades: [1] plant-fungal interactions, examining how fVOCs function in biotechnological applications and ecological signalling; [2] antimicrobial properties of fVOCs from endophytic fungi, emphasising their utility in pathogen control and protective role within host plants; and [3] interactions below ground mediated by microbial VOCs, highlighting how these compounds support communication among soil organisms such as bacteria, fungi, and plant roots.

Furthermore, the data revealed a correlation between the number of citations and co-citation frequency, where highly cited papers, such as those by Morath et al. (Table 3, Pos. 1) and Strobel et al. (Table 3, Pos. 2), also exhibited many co-citations and strong link strengths. This suggests that these influential papers are not only frequently referenced independently of each other but are also commonly cited together, highlighting their central roles within specific research clusters. Additionally, a metric for co-citation per year was introduced, offering insights into the ongoing relevance of these works. It showed that articles with higher co-citations per year tend to be more consistently influential over time, maintaining their impact in the evolving landscape of fVOC research. Morath et al., for instance, although one of the more recent publications, had a notably high co-citation per year count of 7.63 (Table 3, Pos. 1). This rate was significantly higher compared to older works, such as Strobel et al. (Table 3, Pos. 2), which had a lower co-citation per year count of 3.63. Morath et al. (Table 3, Pos. 1) gained substantial attention within a shorter period, underscoring its immediate influence within the field.

Similarly, Effmert et al. (Table 3, Pos. 3), another recent publication, exhibited a relatively high co-citation per year rate of 4.09, compared to older works such as Wheatley et al. (Table 3, Pos. 8), which had a co-citation per year of 1.90. This trend suggests that newer publications, particularly those with high co-citation rates per year, are rapidly establishing themselves as influential within fVOC research.

## Discussion

This study provided a comprehensive overview of research on fVOCs and highlighted their diverse roles in ecological communication, biotechnological applications, and human interactions. Several key themes were identified through a literature review and bibliometric analysis that illustrated the complexity of fVOC research and its impact. These findings emphasised the role of fVOCs in fundamental biological processes and their significant potential for economic and industrial applications, particularly in agriculture, crop science, and industry. Conventional chemical fungicides and pesticides have been widely used in agricultural systems to mitigate losses caused by pathogens and pests. However, their adverse effects on ecosystems are well documented, underscoring the need for alternative solutions, such as fVOCs.

One major concern is the impact of different fungicides on aquatic or soil ecosystems. Studies have shown that they may disrupt critical ecological processes. A study by Jat et al. (2021), for instance, highlighted that the consistent use of some chemicals decreases populations of beneficial soil organisms, such as earthworms and decomposer fungi, which are vital for maintaining healthy soil ecosystems [87].

These findings underscore the urgency of finding sustainable and natural alternatives, such as fVOCs, that reduce pests while preserving ecosystem health and function. Our literature search is also consistent with previous studies identifying the multifunctional nature of fVOCs. the examples of the inhibitory effects of *Trichoderma* spp. VOCs on seed germination [33] and the enhancement of root growth by different fungi VOCs [32] confirm the dual role of these compounds in plant-microbe interactions. They could also serve as an alternative to widely used chemical fertilisers, which can have negative environmental effects, such as soil degradation and enhanced greenhouse gas emissions [88]. Moreover, artificial fertilisers such as nitrogen affect the soil nutrient balance, which, in turn, influences the quality of the end product [89].

Additionally, our findings on the human perception of fungal odours are consistent with the existing literature on the evolutionary basis of odour detection, as demonstrated in the fruit fly *Drosophila melanogaster* [90]. Understanding the evolutionary basis of odour detection across disciplines could provide insights into the human perception of fVOCs and influence the acceptance of certain products or environments. New studies in this field could integrate biological insights with market considerations.

The implications of our study are manifold. Understanding the dual nature of fVOCs in agriculture could lead to innovative pest management strategies and improved crop production. Producing valuable compounds (such as vanillin by biotechnological methods) [53, 91] illustrated the economic potential for the sustainable and scalable production of essential flavours and fragrances. Due to the enormous natural metabolic capacities of fungi, the global market value is estimated at 54.57 trillion USD [92], a number that is expected to increase with new advances and findings in fVOC research.

As our study showed, fVOCs have become crucial for many different research and industry areas over the last two decades, with their research expanding considerably. Early studies (2000–2004), such as Strobel et al. (2001), focused on antimicrobial properties and established their ecological importance in microbial interactions. Research between 2005 and 2010 shifted from understanding the fundamental role of fVOCs to exploring their potential applications, including protection and health-related aspects, as demonstrated by the works by Morath et al. (2012) and Hung et al. (2013).

The range of topics has expanded in recent years (2021–2023) to include sustainable agriculture, with studies on *Trichoderma* and *Fusarium* indicating enhanced plant resilience, as reflected in the work of Venneman and Li [34]. The growing importance of this class of substances for the interaction and communication between organisms has probably contributed to an expansion of biotechnological applications. The economic significance and practical applications of fVOCs have also grown, particularly in agriculture and fragrance production. However, utilising fVOCs in diverse applications flags some unanswered questions, such as the regulatory framework needed to guarantee the safe deployment of fVOCs in industry and agriculture.

While a regulatory basis exists for many fungal organisms, it does not cover the full spectrum of volatile substances they produce. Regulations such as Directive 2000/54/EG in the European Union classify biological agents, including fungal species, according to their hazardous potential, with risk levels ranging from level 2 to level 4 [93]. However, so far, only viral organisms are classified up to level 4. Additionally, handling procedures for organisations and companies are governed by Technical Rules for Biological Agents and Technical Rules for Hazardous Substances in Germany. However, while these guidelines consider fungal metabolites or spores as toxins or sensitising agents, VOCs of biological origin are not specifically addressed. Other regulations specify and regulate chemical substances that may share characteristics with fungal-based VOCs and be produced by them. Many VOCs, however, are generally classified as hazardous substances, even though not all of them might be listed as such.

Further research is needed to establish clear regulations and safe deployment practices for fVOCs in occupational settings to ensure their responsible and effective use by industrial and agricultural stakeholders.

Another critical aspect is climate change, which may negatively impact all agricultural industries. According to a recent study, fungi are likely to continue affecting crops, especially with rising temperatures and changing climate conditions [94]. Moreover, mycotoxin contamination will increase with warmer temperatures, not only weakening host plants but also stimulating more aggressive pathogens, as another recent study suggests [95]. Conversely, climate change is driving the transition to a renewable and bio-based sustainable industry; according to a recent study, fungi could contribute significantly to this transition in many areas due to their complex enzymatic machinery [14]. This again underlines the dual nature of fungi and fVOCs in agricultural and ecological systems.

Additionally, fVOCs are a significant influence on human culture, particularly in culinary contexts. Research into the sensory properties of VOCs in flavours and gourmet foods, such as truffles, gained increasing interest between 2005 and 2015. Studies, such as those by Splivallo et al. (Table 3, Pos. 13), highlighted how fVOCs shape human sensory experiences, aligning with the broader focus on food science within the research cluster. This reflects a sustained interest in the impact of fVOCs on food quality and human perception.

Fungi, particularly filamentous fungi, are key contributors to the production of certain foods, not only due to their nutritional content but also because of the distinctive and often desirable flavours they generate [96]. Furthermore, volatile aroma compounds are essential to the production of blue cheeses by species, such as *Penicillium roqueforti* [97] and traditional foods made with *Aspergillus oryzae*, including soy sauce, miso, and other soybean-based products [98].

While this study offers valuable insights into fVOC research trends and key publications, certain limitations should be acknowledged. Firstly, relying on specific databases (PubMed, Google Scholar, and Scopus) may have restricted the total number of publications included in the analysis. Although these databases provide extensive coverage, relevant studies published in niche journals or languages other than English may have been overlooked, potentially affecting the completeness of emerging topic identification.

Additionally, the keyword combinations used for the literature search (Table 1) and bibliometric analysis may have inadvertently excluded important studies. The specificity required for efficient data retrieval could have led to the omission of research that addresses fVOCs indirectly or within a broader context but lacks explicit tagging with the selected keywords. This limitation may be especially relevant in interdisciplinary or emerging fields within fVOC research, where terminology varies widely.

Finally, while the time period analysed (2000–2023) provided a broad historical perspective, it may underrepresent recent developments, particularly those not yet widely cited. As a result, although the findings accurately reflect well-established trends and influences, they may not fully capture the latest shifts in research focus or emerging innovations still gaining traction. Despite these valuable findings, further research is needed to explore ongoing advancements in this field.

## Conclusions

Our comprehensive research on fVOCs over the past two decades has revealed a transformation from a relatively narrow focus on antimicrobial properties and yeast fermentation into a broad, interdisciplinary field. We analysed 3,738 publications through intensive literature review and bibliometric analysis highlights this thematic diversification, revealing emerging areas of interest, such as indoor air quality, biocontrol, fermentation technology, and microbial community dynamics. Key studies, frequently cited together, serve as intellectual bridges, shaping the progression of the field and fostering cross-disciplinary connections. Research has also diversified to reflect a growing interest in leveraging fVOCs as natural biofungicides that support sustainable agricultural practices. Today, fVOC research is a mature and dynamic field, with insights spanning from molecular mechanisms to ecosystem dynamics and implications reaching from fundamental biology to global markets. As the field continues to expand – encompassing agriculture, environmental health, and food science – fVOCs hold significant potential for developing sustainable, biologically informed solutions to contemporary challenges, including climate change.

## Electronic supplementary material

Below is the link to the electronic supplementary material.

Supplementary Material 1

## Data Availability

No datasets were generated or analysed during the current study.
