# Supplementary material for: A bibliometric analysis of fungal volatile organic compounds
Source: Fungal Biol Biotechnol. 2025 Jul 2;12:12. doi: 10.1186/s40694-025-00203-x (PMC12219455; doi:10.1186/s40694-025-00203-x)
Supplement: Supplementary file 1 — Supplementary Material 1 [file 40694_2025_203_MOESM1_ESM.zip › Supplementary files/20241008_Tab5_MostInfluential Paper.docx]

|  | **Tab. 5.** 20th most influential works based on a co-citation network analysis in VOSviewer. Numbers of co-citation and total link strength directly resulted from the VOSviewer software. The additional information about citations in total and the corresponding DOI was manually added . | | | | | | |
| --- | --- | --- | --- | --- | --- | --- | --- |
| ***Position*** | | ***Publications / Year*** | ***Co-citations*** | ***Co-citations per year (since 2023)*** | ***Citations [Total]*** | ***Total link strength*** | ***DOI*** |
| 1 | | Morath et al. (2012) | 84 | 7.63 | 378 | 56 | <https://doi.org/10.1016/j.fbr.2012.07.001> |
| 2 | | Strobel et al. (2001) | 80 | 3.63 | 435 | 50 | <https://doi.org/10.1099/00221287-147-11-2943> |
| 3 | | Effmert et al. (2012) | 45 | 4.09 | 383 | 35 | <https://doi.org/10.1007/s10886-012-0135-5> |
| 4 | | Mitchell et al. (2010) | 34 | 2.61 | 165 | 25 | <https://doi.org/10.1099/mic.0.032540-0> |
| 5 | | Wenke et al. (2010) | 36 | 2.76 | 213 | 23 | <https://doi.org/10.1007/s00425-009-1076-2> |
| 6 | | Schmidt et al. (2015) | 31 | 3.85 | 324 | 22 | <https://doi.org/10.1038/ismej.2015.42> |
| 7 | | Korpi et al. (2009) | 23 | 1.64 | 404 | 20 | <https://doi.org/10.1080/10408440802291497> |
| 8 | | Wheatley (2002) | 40 | 1.90 | 240 | 20 | <https://doi.org/10.1023/A:1020592802234> |
| 9 | | Ezra et al. (2004) | 23 | 1.21 | 204 | 18 | <https://doi.org/10.1099/mic.0.27334-0> |
| 10 | | Kai et al. (2009) | 20 | 1.42 | 383 | 18 | <https://doi.org/10.1007/s00253-008-1760-3> |
| 11 | | Schulz et al. (2007) | 23 | 1.43 | / | 18 | <https://doi.org/10.1039/B507392H> |
| 12 | | Hung et al. (2013) | 23 | 2.30 | 138 | 17 | <https://doi.org/10.1016/j.funeco.2012.09.005> |
| 13 | | Splivallo et al. (2007) | 22 | 1.38 | 147 | 17 | <https://doi.org/10.1111/j.1469-8137.2007.02141.x> |
| 14 | | Herrmann (2010) | 20 | 1.54 | / | 16 | <https://doi.org/10.1002/9780470669532> |
| 15 | | Matysik et al. (2009) | 26 | 1.86 | 77 | 16 | <https://doi.org/10.1016/j.chemosphere.2009.02.010> |
| 16 | | Combet et al. (2006) | 29 | 1.71 | 206 | 13 | <https://doi.org/10.1007/S10267-006-0318-4> |
| 17 | | Wilkins et al. (2000) | 25 | 1.09 | 119 | 12 | <https://doi.org/10.1016/S0045-6535(99)00273-8> |
| 18 | | Mercier et al. (2004) | 21 | 1.11 | 190 | 10 | <https://doi.org/10.1016/j.postharvbio.2003.08.004> |
| 19 | | Davis et al. (2013) | 23 | 2.30 | 272 | 9 | <https://doi.org/10.1007/s10886-013-0306-z> |
| 20 | | Pretorius et al. (2000) | 30 | 1.30 | 782 | 0 | [https://doi.org/10.1002/1097-0061(20000615)16:8<675::AID-YEA585>3.0.CO;2-B](https://doi.org/10.1002/1097-0061(20000615)16:8%3c675::AID-YEA585%3e3.0.CO;2-B) |
