# Supplementary material for: A bibliometric analysis of fungal volatile organic compounds
Source: Fungal Biol Biotechnol. 2025 Jul 2;12:12. doi: 10.1186/s40694-025-00203-x (PMC12219455; doi:10.1186/s40694-025-00203-x)
Supplement: Supplementary file 1 — Supplementary Material 1 [file 40694_2025_203_MOESM1_ESM.zip › Supplementary files/20241008_vos_cooccurence_pooled.docx]

| **Tab. 4.** 20th most occurred and most cross-linked author keywords among all pooled studies. Total link strength and occurrence have been calculated and measured based on internal VOSviewer software algorithm (see…) | | | | |
| --- | --- | --- | --- | --- |
| ***Number*** | ***Author keyword*** | ***Total link strength*** | ***Occurences*** | ***Cluster*** |
| 1 | volatile organic compounds | 923 | 945 | 1 |
| 2 | yeasts | 405 | 256 | 2 |
| 3 | fermentation | 320 | 192 | 2 |
| 4 | wine | 222 | 120 | 2 |
| 5 | saccharomyces cerevisiae | 216 | 144 | 2 |
| 6 | gc/ms | 197 | 124 | 3 |
| 7 | biological control | 181 | 186 | 1 |
| 8 | aroma | 169 | 101 | 2 |
| 9 | fungi | 167 | 136 | 1 |
| 10 | antifungal activity | 131 | 128 | 3 |
| 11 | solid-phase microextraction | 89 | 54 | 5 |
| 12 | endophytes | 87 | 92 | 1 |
| 13 | non-saccharomyces yeast | 87 | 66 | 2 |
| 14 | flavour | 85 | 50 | 4 |
| 15 | essential oils | 82 | 73 | 3 |
| 16 | bacteria | 75 | 49 | 7 |
| 17 | sensory analysis | 67 | 45 | 2 |
| 18 | antimicrobial activity | 67 | 58 | 3 |
| 19 | trichoderma | 57 | 38 | 1 |
| 20 | botrytis cinerea | 52 | 45 | 1 |
