# Supplementary material for: A bibliometric analysis of fungal volatile organic compounds
Source: Fungal Biol Biotechnol. 2025 Jul 2;12:12. doi: 10.1186/s40694-025-00203-x (PMC12219455; doi:10.1186/s40694-025-00203-x)
Supplement: Supplementary file 1 — Supplementary Material 1 [file 40694_2025_203_MOESM1_ESM.zip › Supplementary files/20241013_publicationsperyear.docx]

| Tab.: Publications per year per main search term | | | | | | |
| --- | --- | --- | --- | --- | --- | --- |
|  | ***Fung*** | ***Mould*** | ***Mushroom*** | ***Yeast*** | ***All*** | *All (cumulative; not shown)* |
| 2023 | 219 | 5 | 30 | 115 | 369 | 3738 |
| 2022 | 194 | 12 | 29 | 109 | 344 | 3369 |
| 2021 | 174 | 6 | 27 | 110 | 317 | 3025 |
| 2020 | 168 | 13 | 20 | 108 | 309 | 2708 |
| 2019 | 124 | 5 | 18 | 92 | 239 | 2399 |
| 2018 | 111 | 2 | 15 | 68 | 196 | 2160 |
| 2017 | 110 | 10 | 8 | 71 | 199 | 1964 |
| 2016 | 109 | 10 | 13 | 41 | 173 | 1765 |
| 2015 | 111 | 12 | 10 | 71 | 204 | 1592 |
| 2014 | 83 | 5 | 9 | 41 | 138 | 1388 |
| 2013 | 67 | 12 | 15 | 44 | 138 | 1250 |
| 2012 | 79 | 13 | 6 | 48 | 146 | 1112 |
| 2011 | 90 | 13 | 6 | 41 | 150 | 966 |
| 2010 | 58 | 13 | 8 | 35 | 114 | 816 |
| 2009 | 56 | 10 | 9 | 43 | 118 | 702 |
| 2008 | 53 | 9 | 6 | 26 | 94 | 584 |
| 2007 | 37 | 8 | 7 | 20 | 72 | 490 |
| 2006 | 45 | 5 | 8 | 30 | 88 | 418 |
| 2005 | 40 | 8 | 1 | 18 | 67 | 330 |
| 2004 | 37 | 3 | 0 | 16 | 56 | 263 |
| 2003 | 27 | 10 | 2 | 20 | 59 | 207 |
| 2002 | 30 | 6 | 2 | 24 | 62 | 148 |
| 2001 | 16 | 7 | 4 | 13 | 40 | 86 |
| 2000 | 27 | 6 | 5 | 8 | 46 | 46 |
| **Sum Pub.** | **2065** | **203** | **258** | **1212** | **3738** |  |
